# Vitamin D reverts resistance to the mTOR inhibitor everolimus in hepatocellular carcinoma through the activation of a miR-375/oncogenes circuit

**DOI:** 10.1038/s41598-019-48081-9

**Published:** 2019-08-12

**Authors:** Donatella Paola Provvisiero, Mariarosaria Negri, Cristina de Angelis, Gilda Di Gennaro, Roberta Patalano, Chiara Simeoli, Fortuna Papa, Rosario Ferrigno, Renata Simona Auriemma, Maria Cristina De Martino, Annamaria Colao, Rosario Pivonello, Claudia Pivonello

**Affiliations:** 1 Dipartimento di Medicina Clinica e Chirurgia, Sezione di Endocrinologia, Università Federico II di Napoli, Naples, Italy; 2 Dipartimento di Sanità Pubblica, Università Federico II di Napoli, Naples, Italy

**Keywords:** Endocrinology, Hepatology

## Abstract

Primary or acquired resistant mechanisms prevent the employment of individualized therapy with target drugs like the mTOR inhibitor everolimus (EVE) in hepatocellular carcinoma (HCC). The current study evaluated the effect of 1,25(OH)_2_Vitamin D (VitD) treatment on EVE sensitivity in established models of HCC cell lines resistant to everolimus (EveR). DNA content and colony formation assays, which measure the proliferative index, revealed that VitD pre-treatment re-sensitizes EveR cells to EVE treatment. The evaluation of epithelial and mesenchymal markers by western blot and immunofluorescence showed that VitD restored an epithelial phenotype in EveR cells, in which prolonged EVE treatment induced transition to mesenchymal phenotype. Moreover, VitD treatment prompted hepatic miRNAs regulation, evaluated by liver miRNA finder qPCR array. In particular, miR-375 expression was up-regulated by VitD in EveR cells, in which miR-375 was down-regulated compared to parental cells, with consequent inhibition of oncogenes involved in drug resistance and epithelial-mesenchymal transition (EMT) such as MTDH, YAP-1 and c-MYC. In conclusion, the results of the current study demonstrated that VitD can re-sensitize HCC cells resistant to EVE treatment triggering miR-375 up-regulation and consequently down-regulating several oncogenes responsible of EMT and drug resistance.

## Introduction

Hepatocellular carcinoma (HCC) is the most common primary liver cancer and the second leading cause of cancer mortality^1^, representing the fifth most common cancer worldwide with 841080 new cases (4.7%), in both sexes, diagnosed in 2018^2^. The main curative options included hepatic resection and liver transplantation, although, in non-eligible cases, different loco-regional therapies are considered as alternative treatment options^1^. Generally, the use of chemotherapeutic compounds is complicated by the pre-existing cirrhotic condition that can perturb the metabolism of chemotherapeutic drugs. Moreover, HCC has been shown to be resistant to the most common chemotherapies^1,3,4^. In the last decades, targeted therapies have entered the field of antineoplastic treatment and have been tested in *in vitro* and *in vivo* research studies but also in several clinical trials, as monotherapy or in combination with additional biological compounds or transcatheter arterial chemoembolization, in the treatment of HCC^5^. Among targeted therapies, the mammalian target of rapamycin (mTOR) inhibitors (mTORi), such as everolimus (EVE), have been evaluated *in vitro*^6^ and *in vivo* as second-line treatment in HCC patients^7^. Unfortunately, drug resistance seems to be the main cause of treatment failure in cancer patients. Recently, epithelial–mesenchymal transition (EMT) has received increasing attention for its role in cancer drug resistance^8^. The modification in gene expression during EMT leads to numerous phenotypic changes, such as cell morphological changes, loss of adhesion and gain of stem cell-like features. The link between EMT and drug resistance has been reported for a long time^8^, but the mechanism is still elusive.

MicroRNAs (miRNAs) are endogenous small non-coding RNAs that regulate gene expression by acting as tumour suppressors or oncogenes, and they are therefore referred to as oncomiRs^9^. miRNAs can regulate drug resistance in cancer cells. Several studies demonstrated a significant correlation between miRNAs dysregulation and drug resistance, suggesting that many miRNAs might be regulators of multiple drug resistance^10–17^. In particular, several miRNAs were found to be up-regulated in HCC cell lines, increasing resistance both to chemotherapeutic agents, such as doxorubicin, cisplatin, paclitaxel, and interferon alpha (IFN-α)/5-fluorouracil, and to molecular targeted drugs, such as sorafenib^11–17^. No data are available regarding miRNA profile in mTORi resistant HCC cells.

The active form of vitamin D, 1,25(OH)_2_Vitamin D (VitD), is a pleiotropic steroid hormone that regulates expression of many genes in several cells, tissues and organs in human. The benefits of VitD on bone are well recognized and VitD supplementation in elderly has been found to be a cost-effective strategy for fracture prevention in the USA and in Western European populations^18–21^. Presently, there are increasing evidences of VitD extra skeletal effects. Indeed, VitD deficiency has been found associated with a variety of diseases, including tumours^22^. In particular, VitD has been described to inhibit *in vitro* proliferation of HCC cell lines^23,24^ and cell growth through decreasing inflammatory cytokine secretion *in vitro* and *in vivo*^25^. Moreover, VitD has been addressed as a new player in the regulation of mTOR pathway^26^ and, recently, several miRNAs have been shown to be regulated by VitD^27–29^.

The aim of the current study was to develop and characterize mTORi resistant HCC cell lines and to demonstrate that VitD is able to revert mTORi resistance by regulating miRNA expression and EMT processes.

## Results

### Characterization of Everolimus resistant (EveR) HCC cell lines

**Figure 1:**
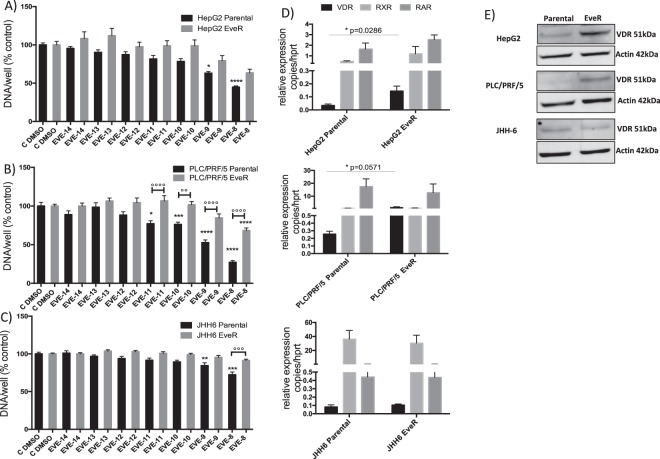
EVE effect on HCC cell proliferation and VDR expression. Cell proliferation evaluated by DNA assay in HepG2 (**A**), PLC/PRF/5 (**B**) and JHH-6 (**C**) cells: parental (black bar) and EveR (gray bar). The data presented are mean ± SEM of three independent experiments. *p < 0.05, **p < 0.01, ***p < 0.001, ****p < 0.0001 vs control; °°p < 0.01, °°°p < 0.001, °°°°p < 0.0001 between groups. (**D**) VDR, RXR and RAR messenger expression, evaluated by RT-qPCR, in HepG2, PLC/PRF/5 and JHH-6 parental and EveR. The data presented are mean ± SEM of three independent experiments. (**E**) VDR protein expression, evaluated with WB, in HepG2, PLC/PRF/5 and JHH-6 parental and EveR.

In order to develop *in vitro* high-level laboratory models resistant to EVE, human HCC cell lines, HepG2, PLC/PRF/5 and JHH-6, were continuously cultured for at least 4 months in presence of EVE 10^−8^ M. The acquired cell resistance was verified by DNA assay after 6 days of treatment with escalating doses of EVE. In all parental and EveR cell lines messenger and protein levels of VitD receptor (VDR) have been evaluated by RT-qPCR and western blot (WB) analyses. PLC/PRF/5 and JHH-6 EveR cells showed a dose-response curve significantly different from the parental cells, particularly at the highest dose of EVE (10^−8^ M) (p < 0.0001, PLC/PRF/5 parental cells treated with EVE 10^−8^ M vs PLC/PRF/5 EveR cells treated with EVE 10^−8^ M; p < 0.001, JHH-6 parental cells treated with EVE 10^−8^ M vs JHH-6 EveR cells treated with EVE 10^−8^ M). In HepG2, no significant difference in cell proliferation inhibition was found between parental and EveR cells, despite it was slightly lower in EveR than in parental cells (Supplementary Fig. 1 and Fig. 1A–C). The analysis of messenger and protein levels of VDR showed that a long-term exposure to EVE increased messenger and protein expression of VDR in HepG2 and PLC/PRF/5 but not in JHH-6 cells (Fig. 1D,E). Since no significant differences in the sensitivity to EVE have been found between parental and EveR HepG2 cells treated with EVE, only PLC/PRF/5 and JHH-6 were selected as models of EveR cells and used for the core study.

### EveR cells display mesenchymal-like markers

**Figure 2:**
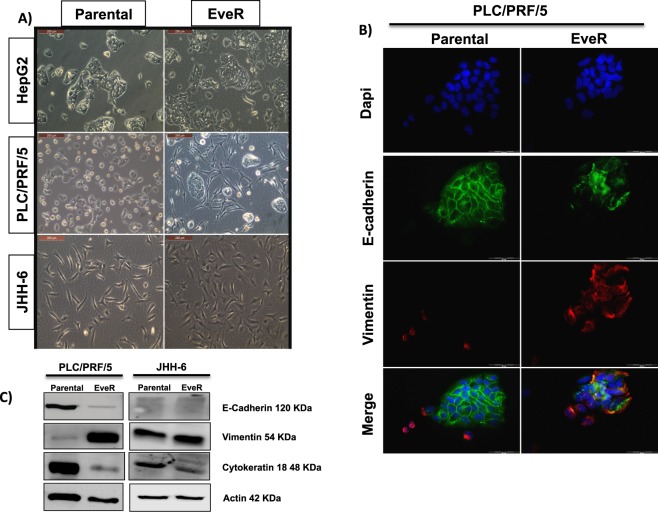
EVE effect on HCC cell morphology and on EMT markers expression in HCC cells. (**A**) Cell morphology evaluation by light microscope, Leica DMIL, at 20x magnification in HepG2, PLC/PRF/5 and JHH-6 parental and EveR. (**B**) E-cadherin (FITC, green) and Vimentin (TRITC, red) protein expression, evaluated by IF, in PLC/PRF/5 parental and EveR using fluorescent microscope Olympus IX51 at 40x magnification. (**C**) E-cadherin, vimentin and cytokeratin 18 protein expression, evaluated by WB, in PLC/PRF/5 and JHH-6 parental and EveR.

The evaluation of EMT in EveR cells was performed by the analysis of cell morphology and epithelial and mesenchymal markers expression. In particular, epithelial and mesenchymal markers in both PLC/PRF/5 and JHH-6 parental and EveR cells were measured by WB, evaluating E-cadherin, cytokeratin 18 and vimentin, and only in PLC/PRF/5 parental and EveR cells by immunofluorescence (IF), evaluating E-cadherin and vimentin. Light microscope use confirmed loosening of cell-cell contact and the acquisition of a spindle-shaped morphology after chronic treatment with EVE (10^−8^ M) in PLC/PRF/5 EveR cells but not in JHH-6 EveR cells (Fig. 2A). According to this morphological result, analysis of epithelial and mesenchymal markers by WB and IF showed a rearrangement of cytoskeletal and cell-cell junction proteins in PLC/PRF/5 cells. Indeed, in PLC/PRF/5 EveR cells, the protein expression of mesenchymal marker vimentin was higher whereas the protein expression of epithelial markers E-cadherin and cytokeratin 18 was lower compared to PLC/PRF/5 parental cells (Fig. 2B,C). In JHH-6 cells, WB analysis demonstrated that, although no protein E-cadherin expression was found, the protein expression of cytokeratin 18 was lower and the protein expression of vimentin was higher in JHH-6 EveR cells compared to JHH-6 parental cells (Fig. 2C).

### VitD restores EVE sensitivity in HCC models EveR

**Figure 3:**
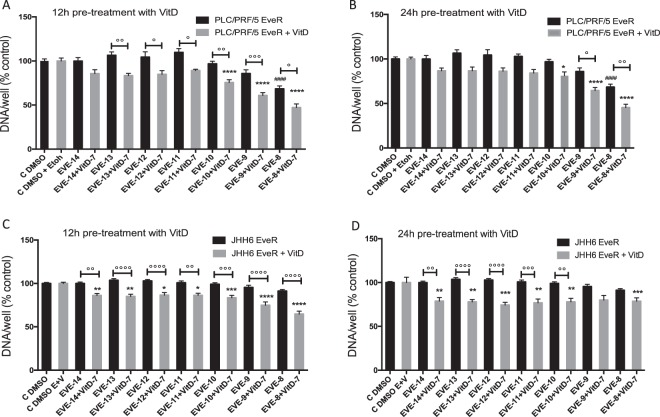
VitD restores EVE sensitivity in HCC EveR cells. Cell proliferation evaluated by DNA assay in PLC/PRF/5 and JHH-6 EveR after 12 and 24 hrs of pre-treatment with VitD and 6 days of co-treatment with EVE. The data presented are mean ± SEM of three independent experiments. ^####^p < 0.0001 vs control plus DMSO; *p < 0.05, **p < 0.01, ***p < 0.001, ****p < 0.0001 vs control plus DMSO and EtOH; °p < 0.05, °°p < 0.01, °°°p < 0.001, °°°°p < 0.0001 between groups.

**Figure 4:**
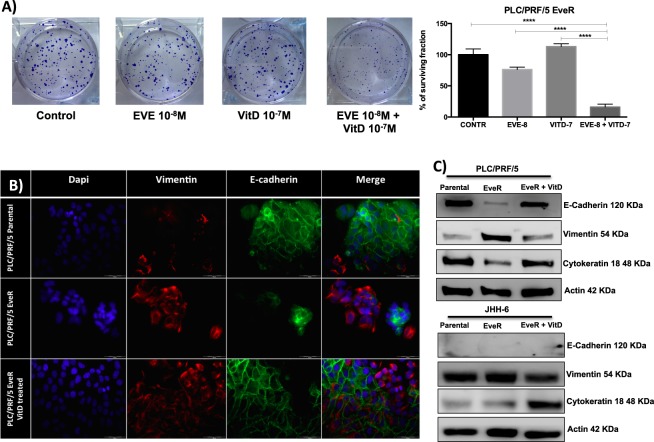
VitD restores EVE sensitivity in HCC EveR cells. (**A**) VitD treatment induces inhibition of colony formation (surviving fraction) in PLC/PRF/5 EveR cells after 24 hrs of pre-treatment and 21 days of co-treatment with EVE. The data presented are mean ± SEM of three independent experiments. ****p < 0.0001 EVE 10^−8^ M + VitD 10^−7^ M vs control, ****p < 0.0001 EVE 10^−8^ M + VitD 10^−7^ M vs VitD 10^−7^ M, ****p < 0.0001 EVE 10^−8^ M + VitD 10^−7^ M vs EVE 10^−8^ M. VitD restores EMT markers expression in HCC EveR cells. E-cadherin (**B**,**C**) and cytokeratin 18 (**C**) expression in PLC/PRF/5 EveR and JHH-6 EveR cells after 6 days of VitD treatment. In IF staining: blu represents DAPI staining of the nuclei; red represents TRITC staining of vimentin protein; green represents FITC staining of E-cadherin protein. All the pictures were captured at 40X magnification.

In order to investigate whether VitD has a role in increasing the cytostatic EVE effect in parental cells or in enhancing the sensitivity to EVE in EveR cells, cell proliferation assay was performed in PLC/PRF/5 and JHH-6 parental and EveR cells and colony formation was also evaluated in PLC/PRF/5 EveR cells, that, contrary to JHH-6 cells, were able to form colonies. No additive effect of VitD and EVE was observed in both PLC/PRF/5 and JHH-6 parental cells (Supplementary Fig. 2). On the other hand, VitD restored EVE sensitivity in both PLC/PRF/5 and JHH-6 EveR cells. In particular, in PLC/PRF/5 EveR cells, EVE induced significant inhibition of cell proliferation only at 10^−8^ M (31,57%, p < 0.0001), whereas a significantly increased cell proliferation inhibitory response to EVE, at the different concentrations, was observed in PLC/PRF/5 EveR cells after 12 or 24 hrs of VitD pre-treatment and 6 days of co-treatment in comparison to PLC/PRF/5 EveR cells treated with EVE as single agent (minimum effect: 19,49Δ%, p < 0.05 in VitD + EVE 10^−12^ M vs EVE 10^−12^ M; maximum effect: 24,91Δ%, p < 0.001 in VitD + EVE 10^−9^ M vs EVE 10^−9^ M) (Fig. 3). In JHH-6 EveR cells, EVE was not able to inhibit cell proliferation at any concentrations, whereas a significantly increased cell proliferation inhibitory response to EVE, at the different concentrations, was observed in JHH-6 EveR cells after 12 or 24 hrs of VitD pre-treatment and 6 days of co-treatment in comparison to JHH-6 EveR cells treated with EVE as single agent (minimum effect: 13,89Δ%, p < 0.01 in VitD + EVE 10^−14^ M vs EVE 10^−14^ M; maximum effect: 26,53Δ%, p < 0.0001 in VitD + EVE 10^−8^ M vs EVE 10^−8^ M) (Fig. 3). In addition, the evaluation of colony formation showed that in PLC/PRF/5 EveR cells, EVE 10^−8^ M and VitD did not induce a significant inhibition of cell surviving fraction when administered alone, whereas 24 hrs of pre-treatment with VitD and 21 days of co-treatment with VitD + EVE resulted in reduction of cell surviving fraction (84,18%) significantly higher than control (p < 0.0001), single treatment with EVE (60,32Δ%; p < 0.0001) and single treatment with VitD (97,24Δ%; p < 0.0001), confirming no major individual effect of VitD when administered alone (Fig. 4A).

### VitD regulates the mesenchymal-epithelial transition (MET) in HCC model EveR

EMT is a reversible mechanism that can underlie drug resistance^8^. In the current study, the effect of VitD on mesenchymal versus epithelial transition has been investigated by exploring the different expression of epithelial and mesenchymal markers in both PLC/PRF/5 and JHH-6 parental and EveR cells, and in EveR cells after a treatment with VitD 10^−7^ M for 6 days, by WB, evaluating E-cadherin, cytokeratin 18 and vimentin, and only in PLC/PRF/5 parental and EveR cells, and in EveR cells after a treatment with VitD for 6 days by IF, evaluating E-cadherin and vimentin. WB analysis demonstrated that in PLC/PRF/5 EveR cells prolonged treatment with VitD induced mesenchymal-epithelial transition increasing the expression of epithelial markers E-cadherin and cytokeratin 18 and reducing expression of mesenchymal marker vimentin compared to untreated PLC/PRF/5 EveR cells, restoring an expression pattern similar to that observed in PLC/PRF/5 parental cells. This effect was also confirmed by IF analysis in PLC/PRF/5 (Fig. 4B,C). Similarly, WB analysis demonstrated that in JHH-6 EveR cells prolonged treatment with VitD increased cytokeratin 18 and reduced vimentin expression compared to untreated JHH-6 EveR cells, restoring an expression pattern similar to that observed in JHH-6 parental cells, although E-cadherin was not found to be expressed either in parental or in EveR cells (Fig. 4C).

### miRNA-375 is down-regulated in HCC EveR cells and its expression is in turn controlled by VitD

**Figure 5:**
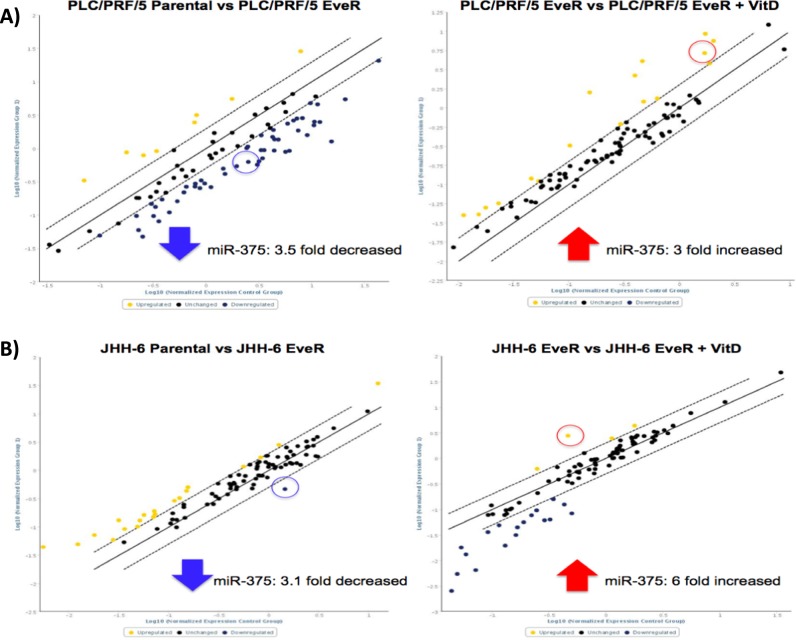
Fold regulation of human liver miRNAs in HCC cells. (**A**) PLC/PRF/5 Parental vs PLC/PRF/5 EveR and PLC/PRF/5 EveR vs PLC/PRF/5 EveR + VitD; (**B**) JHH-6 Parental vs JHH-6 EveR and JHH-6 EveR vs JHH-6 EveR + VitD. Generated scatter plot compares the normalized expression of every miRNA on the array between two groups by plotting them against one another to quickly visualize large miRNA expression changes. The central line indicates unchanged miRNAs expression. The lateral dotted lines represent the selected boundaries. miRNAs whose expression changes are greater than the selected boundaries will be listed as significant up- or down- regulated miRNAs. Yellow dots represent up-regulated miRNAs while blue dots the down-regulated miRNAs. Dot in the circle represents miR-375.

In order to identify the role of miRNAs in drug resistance and their regulation by VitD, liver-specific miRNAs expression has been investigated by MiScript miRNA PCR Arrays Human Liver miFinder kit in PLC/PRF/5 and JHH-6 parental cells, EveR cells and EveR cells after treatment with VitD for 12 hrs. Array analysis revealed that among the different miRNAs significantly regulated in both cell lines, miR-375 showed a typical behaviour. Indeed, in both EveR cell lines miR-375 expression was down-regulated compared to parental cells (PLC/PRF/5 parental cells vs EveR cells: 3.5 fold decreased, p < 0.05; JHH-6 parental cells vs EveR cells: 3.1 fold decreased, p < 0.05); conversely, EveR cells treated with VitD for 12 hrs showed up-regulated expression of miR-375 compared to EveR cells (PLC/PRF/5 EveR cells vs PLC/PRF/5 EveR cells + VitD: 3 fold increased, p < 0.05; JHH-6 EveR cells vs JHH-6 EveR cells + VitD: 6 fold increased, p < 0.05). Fold regulations of miRNAs, including miR-375, have been reported in Supplementary Tables 1 and 2. Regulated miRNAs between parental cells vs EveR cells and EveR cells vs EveR cells + VitD groups were showed in Fig. 5A,B.

### Bioinformatic prediction of miR-375 targets

**Figure 6:**
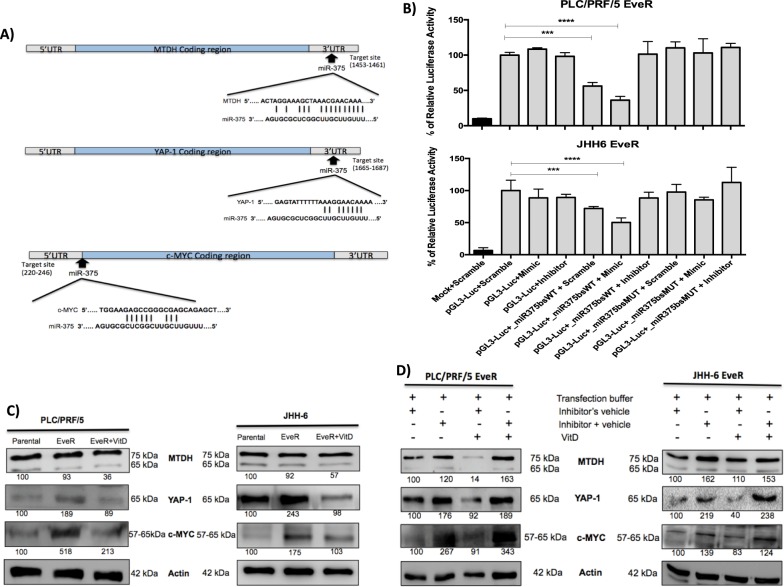
(**A**) Gene structures of MTDH, YAP-1 and c-MYC showing the predicted target site of miR-375. Oncogenes regulation by miR-375. (**B**) Luciferase report assay in PLC/PRF/5 EveR and JHH-6 EveR cells showing that c-MYC is target of miR-375. The data presented are mean ± SEM of three independent experiments. ****p < 0.0001; ***p < 0.001. (**C**) VitD reduces MTDH, YAP-1 and c-MYC protein expression after 6 days of treatment in PLC/PRF/5 EveR and JHH-6 EveR cells. (**D**) Inhibition of miR-375 induced MTDH, YAP-1 and c-MYC protein up-regulation in both EveR cells. The same proteins, instead, were down-regulated in presence of VitD and vehicles. Conversely, MTDH, YAP-1 and c-MYC proteins were up-regulated in presence of both miR-375 inhibitor and VitD. These effects are particularly visible in PLC/PRF/5 EveR cells.

To identify the potential target genes of miR-375, several bioinformatics tools were used. Bioinformatic prediction, determined using the algorithms miRTarBase, TargetScan and miRPath Diana Tools, showed 3′UTR sequences of metadherin (MTDH) and Yes Associated Protein 1 (YAP-1) as putative target sites of miR-375 (Fig. 6A). Both genes have been shown to promote cancer progression and development and to be involved in drug resistance^30,31^. Moreover, MTDH and YAP-1 have been previously experimentally validated by reporter assay, qPCR, WB, and microarray experiments. Additionally, the proto-oncogene c-MYC, involved in drug resistance of several solid and non-solid tumours^32–37^, has been predicted only by miRTarBase but its regulation by miR-375, which has never been previously experimentally validated, is described for the first time in the current study (Fig. 6A).

### c-MYC is a direct target of miR-375

To verify whether c-MYC is a direct target of miR-375, the plasmid pGL3-control encoding for the luciferase reporter gene under the control of a strong promoter, has been engineered to generate the pGL3-Luc-miR375bs-WT and pGL3-Luc-miR375bs-MUT vectors, harboring the wild type or the mutant miR-375 seed sequence of c-MYC, respectively. Luciferase reporter assay indicated that miR-375 directly suppress c-MYC messenger. Indeed, the presence of the wild-type miR-375 seed sequence of c-MYC led to about 40% and 30% reduction of the luciferase activity in PLC/PRF/5 EveR cells and JHH6 EveR cells, respectively, due to the activity of the endogenous miR-375. Moreover, the addition of miR-375 mimic resulted in a strong inhibition of the luciferase activity only in presence of the seed (in PLC/PRF/5 EveR cells 63,77%, p < 0.0001; in JHH6 EveR cells 49,69%, p < 0.001), confirming that the miRNA of interest is able to target and down-regulate the c-MYC messenger (Fig. 6B).

### MTDH, YAP-1 and c-MYC regulation by VitD in HCC EveR cells

Since miR-375 is strongly reduced in EveR cells and increased in VitD treated EveR cells, down-regulation of miR-375 target genes, MTDH, YAP-1 and c-MYC, were evaluated in VitD treated EveR cells. To determine whether 6 days of VitD treatment was able to reduce MTDH, YAP-1 and c-MYC protein expression, WB analysis was performed in PLC/PRF/5 and JHH-6 parental cells, PLC/PRF/5 and JHH-6 EveR cells and in PLC/PRF/5 and JHH-6 EveR cells after VitD treatment. In both PLC/PRF/5 and JHH-6 cells, YAP-1 and c-MYC were strongly up-regulated in EveR cells compared to parental cells while their expression was down-regulated after VitD exposure in both cell lines. Despite a non-significant up-regulation of MTDH in PLC/PRF/5 and JHH-6 EveR cells, VitD treatment promoted MTDH down-regulation in both EveR cells **(**Fig. 6C**)**. To confirm that the regulation of MTDH, YAP-1 and c-MYC was due to a direct-targeted action of miR-375, which is in turn regulated by VitD, EveR cells were transfected with miR-375 inhibitor. In PLC/PRF/5 EveR cells, the use of miR-375 inhibitor expectedly prompted the up-regulation of MTDH, YAP-1 and c-MYC, confirming that these oncogenes are direct target genes of miR-375. Conversely, VitD treatment, in absence of miR-375 inhibitor, induced MTDH, YAP-1 and c-MYC down-regulation. Consistently, in presence of miR-375 inhibitor, VitD was no longer able to down-regulate MTDH, YAP-1 and c-MYC, therefore definitely corroborating the hypothesis that its effect was dependent on miR-375. Similar results, although less evident, were found in JHH-6 EveR cells. Indeed, in JHH-6 EveR cells, the use of miR-375 inhibitor prompted the up-regulation of MTDH, YAP-1 and c-MYC. Conversely, VitD treatment, in absence of miR-375 inhibitor, induced MTDH, YAP-1 and c-MYC down-regulation, whereas, in presence of miR-375 inhibitor, VitD was no longer able to down-regulate MTDH, YAP-1 and c-MYC, corroborating the hypothesis that VitD effect is miR-375 dependent also in this HCC cell model (Fig. 6D).

## Discussion

The results of the current study demonstrated for the first time that VitD restores EVE sensitivity in HCC cells, which had acquired resistance to EVE, through different mechanisms, involving the control of EMT and the regulation of several oncogenes, including MTDH, YAP-1 and c-MYC, by means of the activation of miR-375. Additionally, for the first time, the oncogene c-MYC has been demonstrated to be a relevant target gene of miR-375, and therefore, an indirect target of VitD.

Drug resistance is one of the main causal factors of treatment failure and, consequently, disease relapse and progression in different types of cancer, including HCC. Several molecular mechanisms seem to be involved in the regulation of drug resistance and emerging evidences have revealed that cancer cells undergo EMT that promotes cell survival, aggressiveness and drug resistance^8^. In last years, among the mechanisms underlying drug resistance, miRNAs have emerged and demonstrated to play a role in the regulation of this process in several cancers, including HCC^11–17^.

HCC are poorly responsive to chemotherapy making mandatory searching for novel therapeutic options. Presently, mTORi have been evaluated in phase I/II studies showing activity in patients with advanced or recurrent HCC^38^. However, the phase III study EVOLVE1 demonstrated that EVE treatment did not improve overall survival compared to placebo in patients with advanced HCC intolerant or resistant to sorafenib, a multikinase inhibitors used as first line medical therapy in advanced HCC^39^. The lack of EVE effectiveness in this clinical study can be addressed to several drug resistance mechanisms that trigger the loss of cell sensitivity. Therefore, the elucidation of the molecular mechanisms underlying mTORi resistance is crucial to define whether the mTORi might play a role in the treatment of patients with HCC.

VitD is a steroid hormone commonly found at inadequate levels in the healthy general population and, in particular, in cancer patients; therefore, VitD deficiency or insufficiency represents a widespread phenomenon. The role of VitD on human bone metabolism is very well known and experimental evidence has also suggested a possible association between VitD insufficiency or deficiency and cancer risk^40,41^; however, its influence on the regulation of drug resistance mechanisms remains largely obscure and unexplored.

In the current study, the HepG2, PLC/PRF/5 and JHH-6 cells has been treated at least for 4 months with EVE obtaining a high-level laboratory model of EVE resistant cells^42^. The reversibility of EVE resistance induced by the discontinuation of drug treatment after a long-term exposure has been demonstrated in different cancer cell models^43^; therefore, in the attempt to ensure persistence of EVE resistance, EveR cell lines were treated with EVE during the entire experimental period. The findings of the current study indicate that, in HCC cell lines, after developing resistance to EVE, VDR messenger and protein can be overexpressed and that VitD treatment re-confers sensitivity to EVE, with consequent re-acquisition of EVE ability to inhibit cell proliferation and colony formation. Although VDR overexpression was found in two of the three EveR cell lines, the increase VDR expression seemed not to be correlated with the effect of VitD in EveR cell lines. Indeed, an increased expression of messenger and protein VDR was present in PLC/PRF/5 EveR but not in JHH-6 EveR cell line but in both EveR cell lines the use of VitD efficiently re-sensitized to EVE treatment. On the other hand, in HepG2 EveR cells, a relevant increase in messenger and protein VDR expression, despite an obvious trend in cell resistance after EVE treatment, did not correspond to a significant increase in EVE sensitivity; therefore, HepG2 EveR cell line was not considered to assess the role of VitD in condition of EVE resistance.

EMT and miRNA expression regulation have been explored as potential mechanisms by which VitD controls EVE sensitivity. EMT is a typical feature of epithelial cancer cells undergoing to loss of their apico-basal polarity and acquisition of mesenchymal features, typically accompanied by an increased vimentin expression and a decreased E-cadherin and cytokeratin 18 expression. As expected, EveR acquired a more mesenchymal-like phenotype compared with the parental cell lines. Intriguingly, the findings of the current study demonstrated that this mesenchymal-like phenotype might be reverted to an epithelial-like phenotype by VitD treatment, by increasing E-cadherin and cytokeratin 18 and reducing vimentin protein expression. Indeed, according with previous evidence in colon, breast, prostate and non-small cell lung cancers, VitD can directly increase gene and, consequently, protein expression, of epithelial markers, in particular of components of cell adhesion structures^44^.

It is well established that in mammals miRNAs can regulate gene expression by binding their seed sequence to specific sites in 3′UTR of target genes. In the recent years, increasing evidence pointed out the indication that miRNAs can also bind sites located in 5′UTR and in coding region^45,46^. In particular, it came out that sites located in the coding region are more potent in inhibiting translation, while sites located in the 3′UTR are more efficient in triggering mRNA degradation^47^. However, containing several regulatory elements, such as RNA binding proteins, open reading frames and up-stream start codons, 5′UTR has been recognized as a major feature for gene translation regulation. Therefore, target sequences in 5′UTR can play an important role in miRNA-mediated gene regulation^48^. Mounting evidences indicate that VitD can modulate miRNAs expression levels as demonstrated in cell cultures, animal models and human cohorts; however, the VitD-dependent molecular pathways of miRNA regulation require further investigation^49^. The results of the current study demonstrated that the regulation of a specific miRNA expression was common to both PLC/PRF/5 and JHH-6 cell lines. Indeed, in both HCC EveR cells miR-375 expression was lower than in HCC Eve-sensitive parental cells, and, interestingly, VitD treatment of HCC EveR cell lines induced miR-375 up-regulation compared to VitD-untreated HCC EveR cell lines, restoring in PLC/PRF/5 EveR the miR-375 levels observed in PLC/PRF/5 parental cells or increasing in JHH-6 EveR the miR-375 levels that become closer to that observed in JHH-6 parental cells. The down-regulation of miR-375 has been demonstrated in several cancers^50–52^, including HCC^11,53^, suggesting its tumour-suppressor role. The loss of miRNA-375 leads to increased cell proliferation, invasion and metastatic potential, clinically evolving in a scant cancer survival rate^52^. Emerging evidences are demonstrating that miRNAs are involved in drug resistance and EMT, as key modulators, in many types of cancers. The results of the current study demonstrated that, in EveR cells, VitD dependent miR-375 up-regulation leads, in turn, to down-regulation of several oncogenes, particularly MTDH and YAP-1, selected by using predictive softwares to be potential miR-375 targets involved in drug resistance. MTDH, also known as astrocyte elevated gene-1 and lysine-rich carcinoembryonic antigen-related cell adhesion molecule-1-associated protein, and YAP-1 have been confirmed as targets of miR-375 in previous studies^54,55^. Moreover, the results of the current study demonstrated, for the first time, that c-MYC is also a target of miR-375, since it contains a target site of the miR-375 seed sequence in the 5′UTR (target site in exone 1, lenght 220–246). This result is in agreement with several previous studies showing the existence of target sites in 5′UTR and in coding region of several target genes, but the fact that the majority of the miRNA target prediction tools focus on the 3′UTR has limited the discovery of these alternative miRNA-binding sites. MTDH^56^ as well as YAP-1^57^ and c-MYC^58^ expression was significantly correlated with pathological grade, distant metastasis and poor overall survival in HCC. Furthermore, these oncogenes have been found to be involved in drug resistance^30–32^ and implicated in the regulation of EMT in several cancers. Indeed, in different cancers, positive correlations between MTDH and YAP-1 expression and mesenchymal biomarkers and, on the contrary, negative correlations between MTDH and YAP-1 expression and epithelial biomarkers have been established^59,60^; alike, the up-regulation of c-MYC confers a more mesenchymal features in several cancers^61^. Moreover, the results of the current study demonstrated that VitD can indirectly modulate MTDH, YAP-1 and c-MYC expression by acting through the up-regulation of miR-375 in condition of drug resistance.

Certainly, the use of exclusively experimental *in vitro* models represents a limitation, however, the current study, firstly identified the role of VitD in mTORi resistance in HCC, giving the basis for future *in vivo* preclinical studies evaluating VitD role on mTORi resistant models, i.e. xenografts of HCC cell lines or patients-derived HCC tissue in BALB/c nude mouse recipients.

In conclusion, the results of the current study identified a molecular circuit in which VitD regulates EMT process and reduces oncogene expression, through the up-regulation of miR-375 expression, therefore re-inducing EVE sensitivity, in HCC EveR cell lines. In particular, among the oncogenes, for the first time, c-MYC has been recognized as new target of miR-375. These results suggested a new role of VitD in the field of drug resistance, permitting to hypothesize a new strategy to overcome the mTORi resistance in the treatment of HCC.

## Materials and Methods

### Compounds

Powder of VitD (1,25(OH)_2_Vitamin D, calcitriol) and EVE were supplied by Selleck Chemicals (UK) and dissolved in EtOH and DMSO100%, respectively. Aliquots were stored at −80 °C and fresh aliquots were defrosted prior of each new experiment. Serial dilutions were prepared using EtOH and DMSO 40% reaching vehicles concentrations of 0.04% in the final volume in each well. VitD was used at concentration of 10^−7^ M, a concentration broadly below the VitD dose administered in condition of VitD deficiency and generally used in *in vitro* studies^62^. When combined with VitD, EVE was used at concentrations of 10^−8^ M. This concentration is definitely lower than that is generally used for medical therapy in solid human cancers^63^.

### Cell cultures and generation of Everolimus resistant (EveR) cell lines

Human HCC cell lines HepG2, PLC/PRF/5, and JHH-6 were used for the study. HepG2 were obtained from European Collection of Authenticated Cell Cultures (ECACC), PLC/PRF/5 from American Type Culture Collection and JHH-6 were kindly provided by Prof. Giovanni Vitale from Department of Clinical Sciences and Community Health (DISCCO), University of Milan. HepG2 were cultured as previous described^6^; PLC/PRF/5 were cultured in DMEM medium with 10% of Fetal Bovine Serum (FBS), 1 × 10^5^ U/L penicillin and streptomycin, 0.1% Fungizone (Gibco, Italy) and 1% of MEM non-essential Amino Acids 100x (ThermoFisher, Italy). JHH-6 were cultured in Williams’ Medium E supplemented with 10% FBS, 1 × 10^5^ U/L penicillin and streptomycin, 2 mmol/L L-glutamine, and 0.1% Fungizone (Gibco, Italy). EVE resistant cells (EveR) were developed by continuous culture of cell lines in presence of EVE 10^−8^ M for at least four months^42^. Parental cells were cultured in parallel to resistant ones without addition of EVE. All cell lines were grown at 37 °C in a humidified atmosphere with 5% CO_2_. Regular biochemical tests using MycoAlert^TM^ mycoplasma detection kit (Lonza Walkersville, Inc., USA) were performed to verify the mycoplasma free working conditions of cell lines. Cell lines identity was confirmed by short tandem repeat profiling (LGC Standards Cell Line Authentication service).

### Cell proliferation assay

After trypsinization, HepG2, PLC/PRF/5 and JHH-6 cells were plated in 1 ml of complete culture medium in 24-well plates at different density, based on growth curves. For HepG2 parental and EveR 1.5 × 10^4^, PLC/PRF/5 1.5 × 10^4^, PLC/PRF/5 EveR 2 × 10^4^, JHH-6 parental and JHH-6 EveR 1.5 × 10^4^ cells were plated for 6 days, respectively. The plates were then placed in incubator in 5% CO_2_ at 37 °C. After 24 hrs, EVE was added to each well at different concentrations, ranging between 10^−14^ and 10^−8^ M. When treatment with VitD was combined, cells were pre-treated with VitD 10^−7^ M for 12 or 24 hrs and freshly added every 3 days. Controls were vehicle-treated. Plates were further incubated at 37 °C and 5% CO_2_. Medium was changed and compounds were freshly added every 3 days. After 6 days of treatment, cells were harvested for DNA measurement. Measurement of total DNA content, representative for the number of cells, was performed using the bisbenzimide fluorescent dye (Hoechst 33258) (Boehring Diagnostics, La Jolla, CA), as previously described^64^.

### mRNA isolation and RT-qPCR

mRNA isolation was carried out as previously described^6^. mRNA expression pattern of VDR, retinoid X receptor (RXR), retinoic acid receptor (RAR), was assessed by RT-qPCR. Specific sets of primer sequences used in this study were reported in Supplementary Table 3. SYBR Green based RT-qPCR was set as previously reported^65^. The final product was subjected to graded temperature-dependent dissociation to verify that only one product was amplified. Reactions were run in duplicate and each reaction was repeated thrice on a StepOne Plus real-time PCR machine (Applied Biosystems Foster City, CA, USA). The relative expression levels of each transcript analyzed in each sample were normalized using the housekeeping gene hypoxanthine-guanine phosphoribosyltransferase (HPRT).

### miRNA isolation and RT-qPCR array

The miRNAeasy mini kit (Qiagen. Italy) was used to extract total RNA from samples, including small RNAs. Briefly, PLC/PRF/5 and JHH-6, parental and EveR, cell lines were plated into culture dishes and grown until 65–70% confluence. After pre-treatment with VitD 10^−7^ M for 12 and 24 hrs and treatment with EVE 10^−8^ M for 2 hrs, cells were lysed in QIAzol Lysis Reagent and incubated at room temperature for 5 min. After, chloroform was added in each sample to get three phases, from which isolate RNA. After washings with buffers and centrifugations for several minutes, obtained RNA samples were eluted in 50 μl of RNase-free water. Subsequently, complementary DNA (cDNA) was obtained by synthesis of DNA from RNA template, via reverse transcription. In particular, a concentration of 250 ng/μl of total RNA sample, was added to reverse-transcription master mix consisting in: 5x miScript HiSpec Buffer; 10x miScript Nucleics Mix; RNase-free water; miScript Reverse Transcriptase Mix. Once sample was ready, PCR Arrays was performed.

miScript miRNA PCR Arrays Human Liver miFinder (Qiagen, Italy) were applied to screen candidate miRNAs following manufacture instructions. The analysis of the results of the PCR Array was performed using Qiagen software.

### Colony forming assay

PLC/PRF/5 EveR cell lines were plated into six-well culture dishes and cultured in complete medium for 21 days. Cell number plated in each well was equal to 500 cells/mL. After 24 hrs adhesion, cells were treated with VitD 10^−7^ M and EVE 10^−8^ M alone and in combination. In this latter case, cells have been pre-treated with VitD 10^−7^ M for 24 hrs and then treated with EVE 10^−8^ M in presence of the vehicles of the two drugs. Medium and compounds were refreshed every 3 days. After 21 days, formed colonies were stained and counted as previously reported^66^.

### Immunofluorescence staining

IF staining was performed as previously described^65^ with some changes in the protocol for the different antibodies used. The cells were incubated with primary antibodies against vimentin and E-cadherin for 1 hour and half. Then, slides were washed thrice in 0.1% Triton/PBS for 5 min and incubated with the secondary antibodies for 1 hour (Supplementary Table 4). A 4.6-Diamidino-2-phenylindole (DAPI) (Lonza Group Ltd, Basel, Switzerland) staining, diluted in PBS 1X 1:40000, was used to visualized the nuclei. Images were visualized on an inverted microscope Olympus IX51 equipped for fluorescence and phase contrast microscopy (Olympus, Milan, Italy) and were captured at 60X magnification and acquired with Olimpus Digital Camera F-View II (Olympus, Milan, Italy).

### Cell transfection with miR-375 inhibitor

PLC/PRF/5 EveR and JHH-6 EveR cells, were seeded into six-well culture dishes in serum-free medium at 40–50% confluence. After 15 min of incubation, cells were transfected by transfection reagent HilyMax reconstituted by Lipoform Buffer (Dojindo EU), or by reagent HilyMax and miScript miRNA Inhibitor (Anti-hsa-miR-375; Qiagen, USA) reconstituted by RNase-free water following the supplier’s instructions. After 24 hrs of incubation, VitD 10^−7^ M was added for 72 hrs then the medium was changed and VitD 10^−7^ M was added again for other 72 hrs. After 6 days of treatment, cells were harvested for protein extraction.

### Cell lysis and western blot

Cell lysis and WB analysis were performed as previously described^6^. Primary antibodies specific for VDR, vimentin, cytokeratin 18, E-cadherin, c-MYC, MTDH, YAP-1 and actin were probed on nitrocellulose filters for 2 hrs. Peroxidise-conjugated secondary antibodies used were probed on nitrocellulose filters for 1 hour (Supplementary Table 4). Immunoreactive bands were detected by ECL system. After chemiluminescent reaction, the blot was exposed to ImageQuant Las 4000 (GE Healthcare).

### miRNA target prediction

Bioinformatic prediction was determined using the algorithms TargetScan and Diana Tools to predict targets of miRNA-375. Moreover, predicted targets were also confirmed in the experimentally validated microRNA-target interactions database miRTarBase.

### Cloning 3′UTR constructs and luciferase report assay

The plasmid pGL3-Control (Promega, Italy) encoding for the luciferase reporter gene under the control of a strong promoter, has been engineered to generate the pGL3-Luc-miR375bs-WT and pGL3-Luc-miR375bs-MUT vectors, harboring the wild type or the mutant c-MYC miR-375 seed sequence, respectively. The c-MYC sequence of interest (5′UTR, Exone 1, Gene ID 4609) was: 5′ TGGAA**GAGCCG**GG**CGA**GCAGAGCT 3′, (in bold the miR-375 seed). pGL3-Control plasmid has been digested with XbaI enzyme. The following oligonucleotides containing the XbaI site at 5′and 3′-end have been used for cloning purposes.

c-MYC UTR WT, FW: 5′ pCTAGATGGAAGAGCCGGGCGAGCAGAGCTGCT 3′

c-MYC UTR WT, RV: 5′ pCTAGAGCAGCTCTGCTCGCCCGGCTCTTCCAT 3′

c-MYC UTR MUT, FW: 5′ pCTAGATGGAATTTTTTGGTTTGCAGAGCTGCT 3′

c-MYC UTR MUT, RV: 5′ pCTAGAGCAGCTCTGCAAACCAAAAAATTCCAT 3′

A 5′ phosphorylation has been also added to facilitate the ligation. Sense and antisense nucleotides were resuspended in H_2_O to a final concentration of 100 μM. For the annealing, 10 μM oligos have been diluted in annealing buffer (10 mM Tris-HCl, pH 7.5, 0,1 M NaCl, 1 mM EDTA), incubated at 96 °C for 5 minutes and gradually cooled down to room temperature. The ligation reactions were performed with the Rapid DNA ligation kit (Roche, Italy) according to manufacturers’ instructions. After E. coli transformation and construction amplification, plasmid DNA has been extracted by using the Plasmid Plus Maxi Kit (Qiagen, Italy), checked by PCR and by Sanger sequencing. The construct was further amplified, extracted with the GenElute HP Plamid Maxiprep Kit (Sigma Aldrich, Italy) and quantified by using NanoDrop spectrophotometer.

Lipofectamine 2000 has been employed for transfection of PLC/PRF/5 EveR and JHH6 EveR cells. 24 hrs before transfection, 6*10^4^ PLC/PRF/5 EveR and 3*10^4^ JHH6 EveR cells have been seeded in 24-well plates in complete medium. The transfection has been performed using 800 ng/well of pGL3 reporter vectors (either pGL3-Control, pGL3-miR375bs-WT or pGL3-miR375bs-MUT) and 200 ng/well of pRL-TK Luciferase reporter vector, used for normalization purposes. In co-transfection experiments, 5 pmol/well of miR-375-mimic or inhibitor have been used too. The appropriate scramble miRNA has been used as control. The transfection has been performed in serum free medium and medium has been changed 6–8 hrs after transfection. All the experimental points have been performed in triplicates and three independent experiments have been assessed. Cells have been harvested 48 hrs later according to the instruction reported in the Dual-Luciferase Reporter Assay Kit (Promega). The Firefly Luciferase activity values have been normalized on the Renilla-Luciferase ones to obtain the normalized luciferase activity. The average of the normalized luciferase activity has been calculated and expressed as percentage relative to the one measured in the cells transfected with pGL3-Control.

### Statistical analysis

All the experiments were performed in quadruplicates and were replicated three times with the exception of western blot analysis that were replicated two times. All statistical analyses were performed using SPSS and GraphPad softwares. Differences between the treated groups were assessed by ANOVA, followed by a multiple comparative test (Bonferroni, Newman-Keuls or Dunnett’s correction).

## Supplementary information

Supplementary data

## Data Availability

The datasets generated during and/or analysed during the current study are available from the corresponding author on reasonable request.
