# Supplementary material for: Vitamin D reverts resistance to the mTOR inhibitor everolimus in hepatocellular carcinoma through the activation of a miR-375/oncogenes circuit
Source: Sci Rep. 2019 Aug 12;9:11695. doi: 10.1038/s41598-019-48081-9 (PMC6690984; doi:10.1038/s41598-019-48081-9)
Supplement: Supplementary file 1 — Supplementary data [file 41598_2019_48081_MOESM1_ESM.docx]

**Vitamin D reverts resistance to the mTOR inhibitor everolimus in hepatocellular carcinoma through the activation of a miR-375/oncogenes circuit.**

Donatella P. Provvisiero^1*^, Mariarosaria Negri^1*^, Cristina de Angelis^1,2^, Gilda Di Gennaro^1^, Roberta Patalano^1,2^, Chiara Simeoli^1^, Papa F.^1^, Rosario Ferrigno^1^, Renata S. Auriemma^1^, Maria C. De Martino^1^, Annamaria Colao^1^, Rosario Pivonello^1^, Claudia Pivonello^1^.

^1^Dipartimento di Medicina e Chirurgia, Sezione di Endocrinologia, Università di Napoli «Federico II»; Naples 80131, Italy; ^2^Dipartimento di Sanità Pubblica, Università di Napoli «Federico II», Naples, Italy.

*These two authors contributed equally to this work.

**Supplementary Figures and Tables legends**

**Supplementary Figure 1:** Eve effect on cell proliferation evaluated by DNA assay on HCC cell lines parental and EVE resistant *p<0.05, using Friedman’s test corrected with Dunn’s multiple comparison test.

**Supplementary Figure 2:** Eve effect alone and with VitD on cell proliferation evaluated by DNA assay on PLC/PRF/5 (A) and JHH-6 (B) cells: parental (black bar) and parental treated with EVE + VitD (gray bar). *p<0.05, **p<0.01, ***p<0.001, ****p<0.0001 vs control.

**Supplementary Table 1**: Genes whose expression changes are greater than the selected boundaries will be listed as significant up- or down- regulated genes. Table reports the fold regulation of upregulated or down regulated genes, with miR-375 in bold type, in PLC/PRF/5 parental vs EveR and PLC/PRF/5 EveR vs PLC/PRF/5 EveR+VitD.

**Supplementary Table 2**: Genes whose expression changes are greater than the selected boundaries will be listed as significant up- or down- regulated genes. Table reports the fold regulation of upregulated or down regulated genes, with miR-375 in bold type, in JHH-6 parental vs EveR and JHH-6 EveR vs JHH-6 EveR+VitD.

**Supplementary Table 3**: Primers forward and revers used to detect VDR, RAR and RXR by using RT-qPCR.

| **Antibody** | **Manufacturer** | **Code** | **Host species** | **Dilution** | **Application** |
| --- | --- | --- | --- | --- | --- |
| Vimentin | Abcam | ab92547 | Rabbit monoclonal | 1/250 | IF |
| E-Cadherin | Santa Cruz | sc-21791 | Mouse monoclonal | 1/100 | IF |
| Secondary antibody FITC conjugated | Millipore | AP124F | Goat anti-Mouse | 1/500 | IF |
| Secondary antibody TRITC conjugated | ImmunoReagent | Gtx-Rb-003-DRHO | Goat anti-Rabbit | 1/500 | IF |
| Vimentin | Abcam | ab92547 | Rabbit monoclonal | 1/1000 | WB |
| Cytokeratin-18 | Sigma Aldrich | C-1399 | Mouse monoclonal | 1/250 | WB |
| E-cadherin | Santa Cruz | sc-21791 | Mouse monoclonal | 1/500 | WB |
| c-Myc | Cell signalling | #5605 | Rabbit monoclonal | 1/1000 | WB |
| Lyric/Metadherin | Cell signalling | #9596 | Mouse monoclonal | 1/1000 | WB |
| YAP-1 | Abcam | ab52771 | Rabbit monoclonal | 1/1000 | WB |
| VDR | Santa Cruz | sc13133 | Mouse monoclonal | 1/500 | WB |
| Actin | Sigma Aldrich | A4700 | Mouse monoclonal | 1/10000 | WB |
| Secondary antibody HRP conjugated | ImmunoReagent | Gtx-Mu-003-DHRPX | Goat anti-Mouse IgG | 1/2000 | WB |
| Secondary antibody HRP conjugated | ImmunoReagent | Dkx-Rb-003-DHRPX | Donkey anti-Rabbit IgG | 1/2000 | WB |

**Supplementary Table 4**: Antibodies used for IF and WB analysis.

**Supplementary information: Full gel Images Figure 1E**

**48 kDa**

**63 kDa**

**PC**

**JHH-6**

**Parental**

**EveR**

**PLC/PRF/5**

**Parental**

**EveR**

**HepG2**

**Parental**

**EveR**

**VDR**

**PC**

**JHH-6**

**Parental**

**EveR**

**PLC/PRF/5**

**Parental**

**EveR**

**HepG2**

**Parental**

**EveR**

**63 kDa**

**Actin**

**48 kDa**

**Supplementary information: Full gel Images Figure 2C**

**48 kDa**

**35 kDa**

**Actin**

**PLC/PRF/5**

**Parental**

**EveR**

**63 kDa**

**48 kDa**

**PLC/PRF/5**

**Parental**

**EveR**

**Vimentin**

**PLC/PRF/5**

**Parental**

**EveR**

**63 kDa**

**48 kDa**

**35 kDa**

**Cytokeratin**

**E-Cadherin**

**PLC/PRF/5**

**Parental**

**EveR**

**100 kDa**

**75 kDa**

**JHH-6**

**Vimentin**

**Supplementary information: Full gel Images Figure 2C**

**E-cadherin**

**Parental**

**EveR**

**Cytokeratin**

**JHH-6**

**JHH-6**

**Parental**

**EveR**

**48 kDa**

**48 kDa**

**EveR**

**Parental**

**100 kDa**

**48 kDa**

**Parental**

**EveR**

**JHH-6**

**Actin**

**Supplementary information: Full gel Images Figure 4C**

**PLC/PRF/5**

**Parental**

**EveR**

**EveR + VitD**

**100 kDa**

**E-Cadherin**

**48 kDa**

**35 kDa**

**PLC/PRF/5**

**Parental**

**EveR**

**EveR + VitD**

**Vimentin**

**PLC/PRF/5**

**Parental**

**EveR**

**EveR + VitD**

**48 kDa**

**Cytokeratin**

**PLC/PRF/5**

**Parental**

**EveR + VitD**

**EveR**

**Actin**

**48 kDa**

**35 kDa**

**Supplementary information: Full gel Images Figure 6C**

**63 kDa**

**48 kDa**

**Parental**

**EveR + VitD**

**EveR**

**c-MYC**

**Parental**

**EveR + VitD**

**EveR**

**MTDH**

**75 kDa**

**63 kDa**

**Parental**

**EveR + VitD**

**EveR**

**Actin**

**48 kDa**

**75 kDa**

**63 kDa**

**Parental**

**EveR + VitD**

**EveR**

**YAP-1**

**PLC/PRF/5**

**Supplementary information: Full gel Images Figure 6C**

**JHH-6**

**63 kDa**

**75 kDa**

**Parental**

**EveR + VitD**

**EveR**

**YAP-1**

**Parental**

**EveR + VitD**

**EveR**

**MTDH**

**75 kDa**

**63 kDa**

**Parental**

**EveR + VitD**

**EveR**

**Actin**

**48 kDa**

**Parental**

**EveR + VitD**

**EveR**

**c-MYC**

**63 kDa**

**Supplementary information: Full gel Images Figure 6D**

**PLC/PRF/5**

**63 kDa**

**75 kDa**

**MTDH**

**63 kDa**

**YAP-1**

**c-MYC**

**48 kDa**

**63 kDa**

**48 kDa**

**ACTIN**

**Supplementary information: Full gel Images Figure 6D**

**JHH-6**

**63 kDa**

**75 kDa**

**MTDH**

**c-MYC**

**63 kDa**

**75 kDa**

**ACTIN**

**48 kDa**

**63 kDa**

**YAP-1**
